# A Reliable Method for the Selection of Exploitable Melanoma Archival Paraffin Embedded Tissues for Transcript Biomarker Profiling

**DOI:** 10.1371/journal.pone.0029143

**Published:** 2012-01-17

**Authors:** Celeste Lebbe, Mickael Guedj, Nicole Basset-Seguin, Marie Pierre Podgorniak, Suzanne Menashi, Anne Janin, Samia Mourah

**Affiliations:** 1 Département de Dermatologie, hôpital saint Louis Paris, Paris, France; 2 Université Paris 7- Denis Diderot, Paris, France; 3 Ligue Nationale Contre le Cancer, Cartes d'Identité des Tumeurs program, Paris, France; 4 AP-HP, Hôpital Saint-Louis, Laboratoire de Pharmacologie-Génétique, Paris, France; 5 CNRS, EAC 7149, Laboratoire CRRET, Université Paris 12, Créteil, France; 6 Inserm, U728, Paris, France; 7 AP-HP, Hôpital Saint-Louis, Laboratoire de Pathologie, Paris, France; 8 Inserm, UMR-S 940, Paris, France; The University of Kansas Medical Center, United States of America

## Abstract

The source tissue for biomarkers mRNA expression profiling of tumors has traditionally been fresh-frozen tissue. The adaptation of formalin-fixed, paraffin-embedded (FFPE) tissues for routine mRNA profiling would however be invaluable in view of their abundance and the clinical information related to them. However, their use in the clinic remains a challenge due to the poor quality of RNA extracted from such tissues. Here, we developed a method for the selection of melanoma archival paraffin-embedded tissues that can be reliably used for transcript biomarker profiling. For that, we used qRT-PCR to conduct a comparative study in matched pairs of frozen and FFPE melanoma tissues of the expression of 25 genes involved in angiogenesis/tumor invasion and 15 housekeeping genes. A classification method was developed that can select the samples with a good frozen/FFPE correlation and identify those that should be discarded on the basis of paraffin data for four reference genes only. We propose therefore a simple and inexpensive assay which improves reliability of mRNA profiling in FFPE samples by allowing the identification and analysis of “good” samples only. This assay which can be extended to other genes would however need validation at the clinical level and on independent tumor series.

## Introduction

Malignant melanoma is one of the most rapidly spreading cancers in terms of worldwide incidence [1]. The lack of prognostic markers or efficient treatments of advanced melanoma represents a major problem in patient management [2], [3]. Melanoma personalized medicine is promising but requires the discovery and application of clear prognostic and predictive biomarkers to guide therapeutic decisions [4]. The gold standard of source tissue for biomarkers mRNA expression profiling has traditionally been fresh-frozen tissue which can be feasible and informative in the evaluation of gene transcripts. However, formalin-fixed paraffin-embedded tissue (FFPE) represents by far the most abundant supply of melanoma tumors and as a rule the sole material available for primary tumors [5], [6]. Indeed, with the enormous amount of data retrievable stored in archived formalin-fixed paraffin-embedded tissue, it will prove invaluable if biomarkers transcript expression levels could be routinely and systematically analyzed in FFPE tissues, particularly for retrospective studies and for the characterization of rare or small tumors. However, their routine use in the clinic has been hampered because of the poor quality of RNA extracted from them. However, a few emerging studies using qRT-PCR as well as microarrays suggested these FFPE samples can be used to validate biomarker signatures associated with clinical features, survival and therapeutic response [7], [8], [9], [10], [11], [12]. These studies, conducted mainly in breast cancer tissues have shown a strong correlation in transcript expression between paired FFPE and frozen tissues which was independent of tissue fixation time and storage in paraffin.

Despite a wealth of data, the most useful prognostic indicators of primary melanoma remain Breslow depth, presence or absence of ulceration, mitotic index for thin tumors and lymph node involvement. Recently, the prognostic value of BRAF and NRAS mutation was demonstrated in several retrospective studies [13], [14] and [Jakob J et al., ASCO 2011]. The importance of targeting this pathway for melanoma treatment has been demonstrated in vitro, in pre-clinical animal models and more recently in recent clinical trials [15], [16], [17]. However the observed response in these trials seems to be transient and only for the 50% of melanoma mutated in BRAF, underlining the need for searching new relevant targets in [18], [19]. In a recent multiparametric study deciphering tumor angiogenesis and invasion in melanoma, we demonstrated that the expression of VEGF 121 and PAI1 was significantly associated with the presence of a micrometastasis in the sentinel lymph node [20] and [Mourah et al, AACR 2007] highlighting the prognostic potential of the genes expressed in these biological pathways.

In order to validate novel biomarkers using FFPE melanoma collections, we conducted a comparative study using qRT-PCR on a wider biomarkers gene panel involved in angiogenesis/tumor invasion in matched pairs of frozen and FFPE melanoma tissues. A statistical method was developed that can select the samples with good correlations and identify those that should be discarded on the basis of the paraffin data only.

## Results

**Figure 1:**
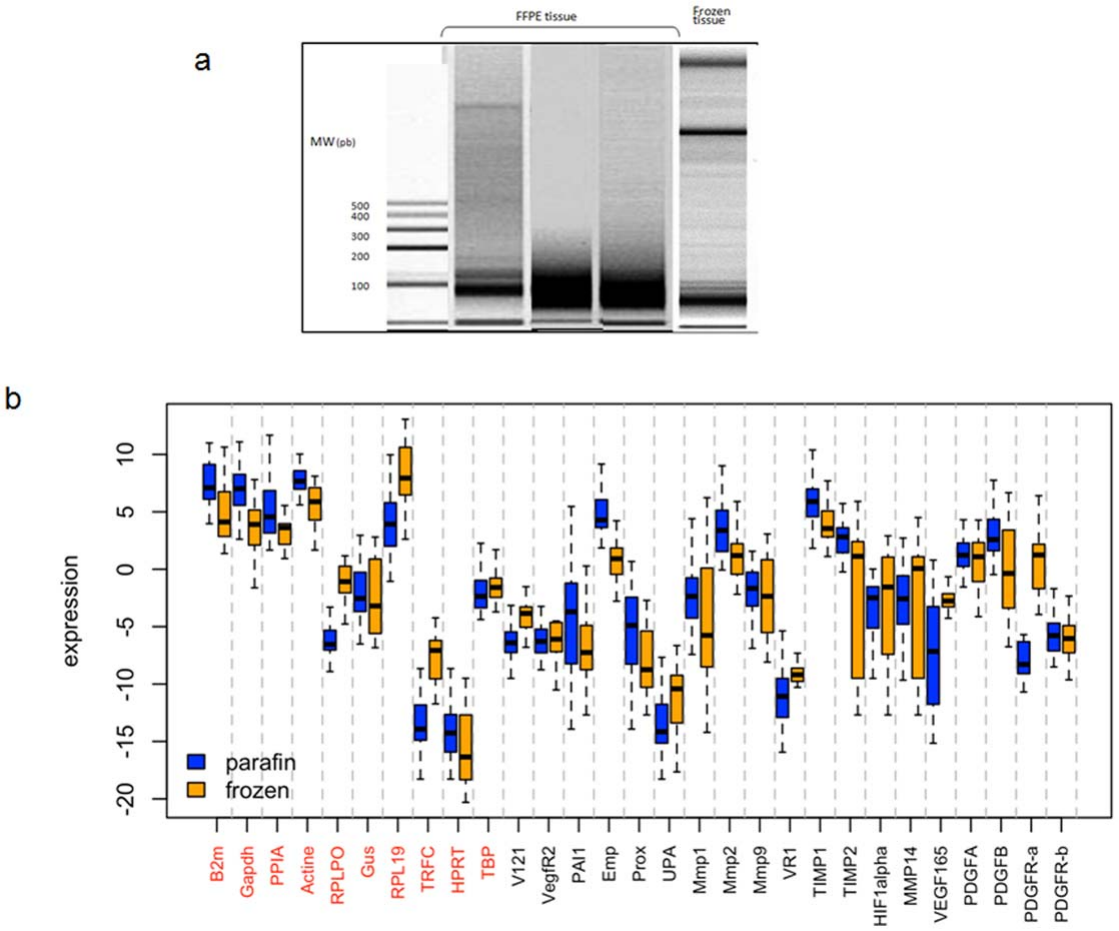
RNA analyses. **a**. Representative total RNA integrity analysis paired frozen and FFPE tissue specimens using Capillary electrophoresis Agilent 2100 bioanalyzer, shows that in the FFPE samples RNA exists primarily as fragments between 200 and 100 bases in length. Left panel: fresh frozen human melanoma tissue. Right panel: matched FFPE tissue. **b**. Boxplot represents the mean mRNA levels and gene expression between frozen and FFPE samples. In red, reference genes.

Comparison of RNA Expression Profiles from FFPE and Fresh Frozen Melanoma Tissues: The expression in malignant melanoma of 25 genes involved in angiogenesis, lymphangiogenesis and tumor invasion pathways was analyzed. For that, total RNA was prepared from 25 matched pairs of frozen and FFPE samples. Inspection of RNA by Agilent Bioanalyzer electrophoresis demonstrated a typical non degraded RNA profile in the frozen specimens while FFPE extracts displayed degraded RNA around 150 and 50 bp, depending on the samples (**Fig. 1a**). These observations are consistent with previous studies which described similar profiles for FFPE.

Heterogeneity in the quality and quantity of the RNA extracted is known to be mainly due to variations in tissue quantity, fixation type and to the delay in tissue fixation after surgery. Furthermore, the efficiency of the reverse transcription and the PCR itself may represent an added variability parameter. In view of this, the qRT-PCR measurements of the genes of interest were normalized to a validated set of housekeeping genes. This validated set was determined by comparing 15 different housekeeping genes between frozen and FFPE matched tissues, out of which 10 genes showing stable expression (very close means between FFPE and frozen specimens) were retained as reference housekeeping gene set.

**Fig. 1b** represents a Boxplot of mean mRNA levels of 19 genes of interest and 10 housekeeping genes in all frozen and FFPE samples showing comparable means for most but not all genes. As example, VEGFR-2 and PDGFR-beta show closely matched means while MMP1 and PDGFR-alpha were unmatched.

**Figure 2:**
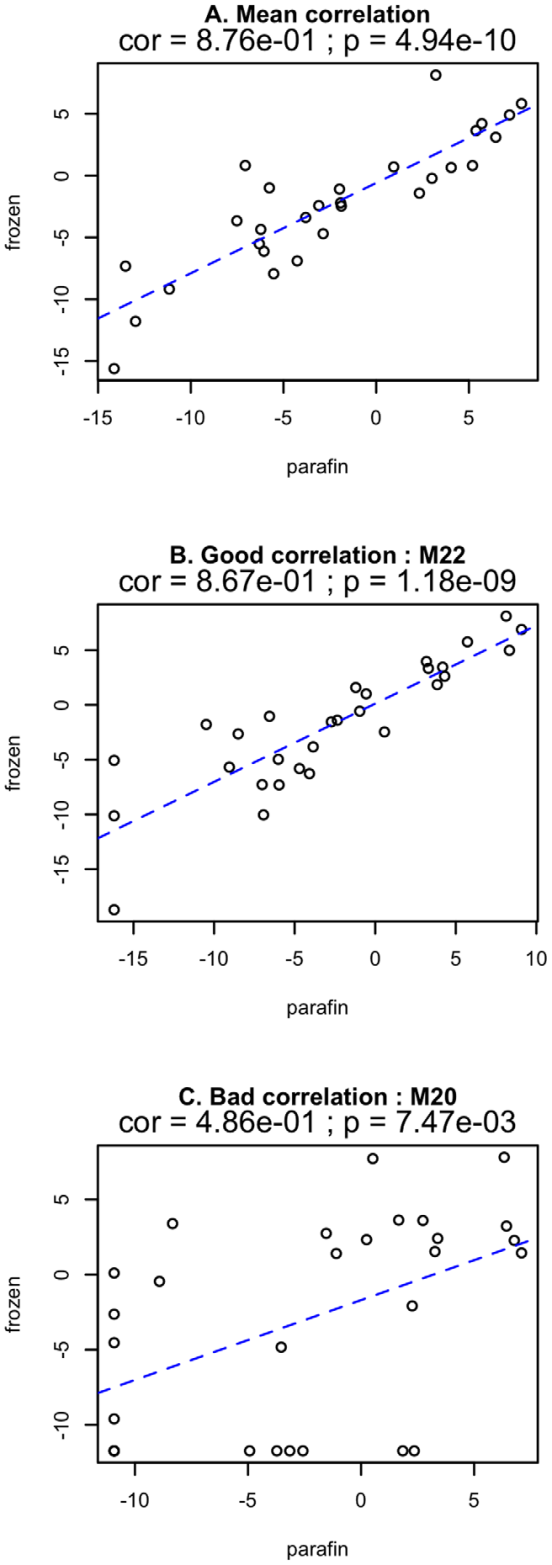
Frozen/FFPE correlations. **a**. Correlation in all the patients and for all the genes between the two tissue preparation methods. The adjusted Pearson correlation between FFPE and frozen tissue for all tested genes was greater (Pearson coefficient = 0.88 p<0,0001). **b**. Two examples of correlations determined individually for each patient measured in all genes.

A correlation frozen/FFPE on the median of each gene was evaluated on all patients, yielding a very good Person correlation coefficient of 0.88, p<0.0001, as presented in **Fig. 2a**.

The same correlation was determined individually for each patient and examples of patients with good, average and bad frozen/FFPE correlation is shown in **Fig. 2b and 2c** (Person correlation coefficient of 0.87 and 0.48, p<0.0001 and p = 0.007 respectively).

**Figure 3:**
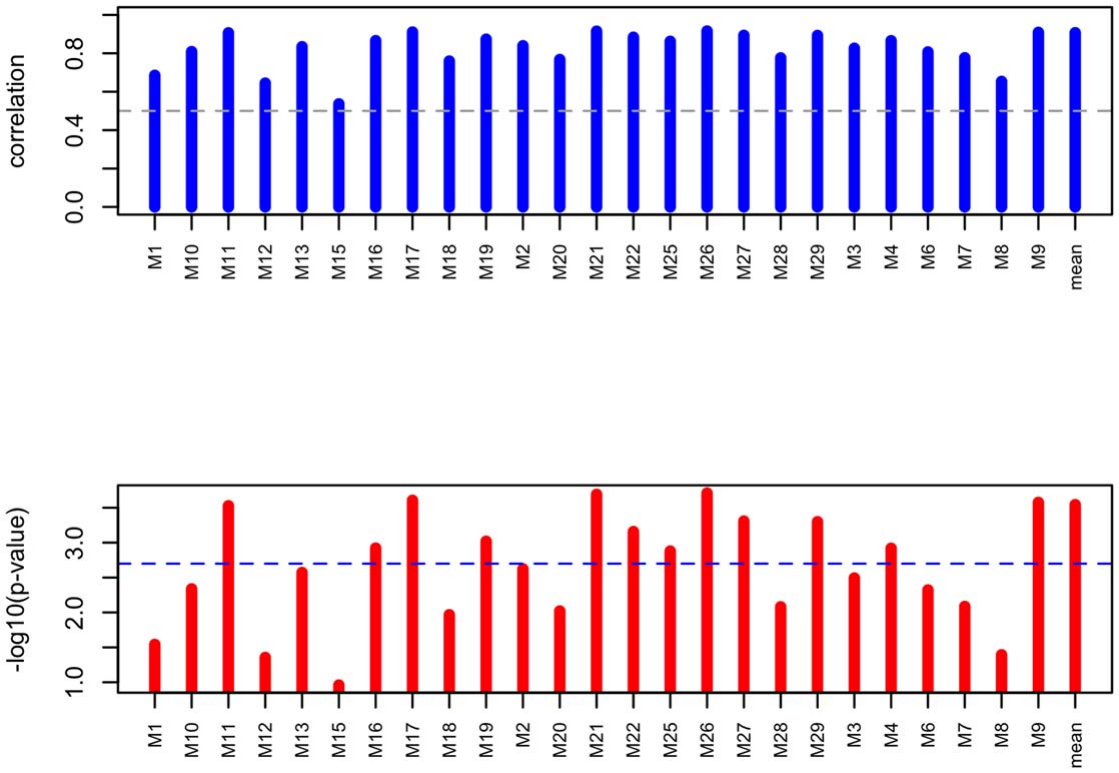
Corrected FFPE/Frozen correlations for each individual. The correlation test tries the hypothesis «the correlation is useless”. The threshold represents the threshold reject alpha = 5% (correct for the multiple test by Bonferroni). The individuals to spread are the ones who are below the threshold.

After Bonferroni adjustment for multiple-testing, we have chosen as ‘good’ the samples with an adjusted p-value below the 5% level and as ‘bad’ those with an adjusted p-value above 10% level. Remaining samples with adjusted p-values between 5% and 10% were considered as ‘average’. Out of the 25 samples analysed, we obtained 21 good, one average and 3 bad samples (**Fig. 3**) (see Table S1).

Identification of samples with a good gene expression correlation between frozen and FFPE: We next aimed to develop a simple statistical method that can identify melanoma samples with a good correlation frozen/FFPE and so to discard samples for which expression levels do not correlate, on the basis of paraffin data for reference genes only.

This approach was based on the assumption that the expression level for these reference genes should remain globally stable across good samples. To this end, we defined ‘Mean-Good-Expression-Profile’ (MGEP) as the mean of individual good samples expression profiles and those samples deviating significantly from the MGEP as bad sample profiles. Deviation was measured and tested with a classical chi-square test. This approach is fully described in Figure S1, and a R script is available on demand.

Among the 21 good samples identified, 14 were used as training samples to construct the MGEP (selected based on a correlation p-value below 10^−3^). The 7 remaining good samples and the 3 bad samples were used for validation.

Of all the possible sets of reference genes available to construct the MGEP, we chose the set with the minimum mean coefficient of variation on the training samples which corresponds to four genes: *Actine, HPRT, TBP and TRFC*. With this set of reference genes, our MGEP-based approach discriminated perfectly the 21 good from the 3 bad samples (100% of good prediction). The remaining average sample was identified as good.

## Discussion

FFPE tumor samples represent a great potential for gene expression profiling. However, their use was so far limited by the poor quality of RNA extracted from such tissues which reduces the reliability of biomarker quantification [21]. This study was conducted on melanoma lesions comparing frozen and FFPE extracts for a panel of 25 genes involved in angiogenesis and invasion. Data analysis revealed that in spite of the degraded RNA in the FFPE samples, only a small proportion of extracts could not be exploited. We therefore propose herein a simple and inexpensive assay which specifically identifies this subgroup which may be discarded thus allowing the selection of exploitable FFPE samples. The fact that this proposed assay is based on the assessment of only 4 reference genes to select “good” melanoma archived samples renders it suitable for clinical use.

Our results show a very good Pearson CC of 0.88, higher than previously reported in the few studies attempting to show the reliability of FFPE RNA extracts in matched FFPE/frozen tissues which at best yielded values between 0.7–0.8 in various tumor types [6], [21], [22]. Several factors may have contributed to the higher Person values obtained in our study i) all samples were monocentrically collected and thus variations due to delay in tissue fixation after surgery as well as fixation time were minimal; ii) all analysed sample blocks were checked by a pathologist to contain at least 90% of tumor cells; iii) transcript quantification used amplicons smaller than 100 bp (between 60 and 80 bp) which allows the amplification of greater number of genes in the degraded specimens.

The high FFPE/Frozen correlation obtained when considering the whole studied melanoma samples nevertheless contained several samples (3/24) with bad correlation (0.4), prompting us to develop a method to distinguish and eliminate these bad samples and so improve reliability of mRNA profiling in FFPE samples. The method described here can therefore contribute to improve validity of prognostic or predictive biomarkers in clinical work.

Cronin et al previously validated both analytically and clinically a FFPE molecular biomarker tests in breast cancer patients predicting survival and therapeutic response [8]. With this method using amplicons of approximately 100 bp for transcript quantification, a 16 gene signature was shown to be enough to predict response to Tamoxifen genes [11]. Our results presented here suggest that discarding individual bad samples in these studies may have further improved the predictive value of transcript biomarkers to response to treatment.

Since the validation of our test is required before it can be recommended for routine use in evaluating prognostic markers in FFPE samples, this pilot study is currently extended to include large cohorts, such as the melancohort already available for the Great Paris area [23]. In addition, this new test could be suitable for identifying new biomarkers involved in other molecular pathways regulating melanoma progression, the inclusion of which will undoubtedly improve the prognostic value of the test.

## Materials and Methods

### Patients

From 2000 to 2005, formalin-fixed and paraffin-embedded (FFPE) tissue specimens or frozen for the same tumor sections were available for 25 patients referred to our department with primary melanoma n = 7, cutaneous metastasis n = 4, lymph node metastasis n = 14. The study was performed in accordance with the precepts established by the Helsinki Declaration and approved by the Hopital Saint Louis Research Ethic Committee (Paris, France). All patients gave informed written consent.

### Samples, RNA extraction and reverse transcription

All tissues were collected according to the guidelines and policies of Saint Louis Hospital – University of Paris 7 Institutional Board. Fresh frozen melanoma tissues were divided and half kept frozen and half fixed in formalin and processed for paraffin embedded. Over 90% of the tissue is composed of tumor cells. Five and ten 10 µm sections for frozen tissue for FFPE block respectively were obtained for the RNA extraction. The total RNA of the 25 pairs of archival melanoma tumor FFPE blocks and matching frozen tumors were extracted with the Chomcynsky and Sacchi method [24] for the frozen specimens and using Qiagen RNA FFPE extraction kit after xylene traitment for FFPE specimens (10 sections of 10 µm) according to the manufacturer's protocol. Each sample was treated with DNase I, to eliminate any traces of genomic DNA. Reverse transcription was performed using Super-Script II (Invitrogen). FFPE tissue RNA analysis and taqMan primer and probe design.

The total RNA yield was determined using a NanoDrop ND-1000 spectrophotometer (NanoDrop Tech, Wilmington, DE). RNA integrity was assessed by Agilent 2100 Bioanalyzer electrophoresis (Agilent Technologies) compared to standard reference RNA as previously described [8].

RT-PCR probes and primers were designed, tested and validated in our laboratory. Amplicon sizes were preferably limited to less than 200 pb in length. Fluorogenic probes were dual-labelled with 5-FAM as a reporter and TAMRA as a quencher.

### Multiparametric transcripts quantification

**The studied angiogenesis/lymphangiogenesis and invasion biomarkers** were VEGFs (solubles forms VEGF121 and VEGF165), VEGF recepteurs (VEGFR1 and VEGFR2), PDGF-A, PDGF-B, PDGF receptors (PDGFR-alpha and –beta), Serine proteases (uPA PAI-1) and Matrix Metalloproteinase (MMP1, 2, 9 and 14), MMP inhibitors(TIMP1 and 2), protease inducer EMMPRIN, regulators transcription factors of angio/lymphangio: HIF1a, PROX-1. **The studied reference transcripts** were TBP, B2 microglobin, GAPDH, ACTBP, GUS, PPIA, TFRC, 18S, 5S, RPLP1, RPLP0, RPL5, RPL19, Actin-alpha and beta-actin.

TaqMan reactions were performed to quantify the multiparametric transcripts. The quantification was using the PerfectProbe Master Mix kit (AnyGenes, France) on a LightCycler 2.0. All the experiements were measured in duplicate. PCR cycling was performed as follows: 95°C for 10 minutes for one cycle, 95°C for 20 secondes, and 60°C for 45 secondes, for 40 cycles.

### Normalization

To compare expression profiles between specimens, normalization based on 15 reference genes was used to correct for differences arising from variability in RNA quality and total quantity of RNA in each assay. 10 reference genes were selected for use from among 15 candidate reference genes tested in this assay. The relative quantification of each transcript was referred to the Cronin work [8].

### Statistical Analysis

The statistical analyses described in the section «Results» was assayed with the software R^1^ (version 2.13.1). P-values are considered significant below the 5% level after Bonferroni adjustment for multiple-testing.

## Supporting Information

Figure S1 Classification approach: supplementary method defining the ‘Mean-Good-Expression-Profile’ (MGEP) as the mean of individual good samples expression profiles and those samples deviating significantly from the MGEP as bad sample profiles. Deviation was measured and tested with a classical chi-square test.
(PDF)

Table S1 Sample correlations and quality.
(XLS)

## References

[1] de Vries E, Coebergh JW (2004). Cutaneous malignant melanoma in Europe.. Eur J Cancer.

[2] Dickson PV, Gershenwald JE (2011). Staging and prognosis of cutaneous melanoma.. Surg Oncol Clin N Am.

[3] Essner R (2006). Sentinel lymph node biopsy and melanoma biology.. Clin Cancer Res.

[4] Nathanson KL (2010). Using genetics and genomics strategies to personalize therapy for cancer: focus on melanoma.. Biochem Pharmacol.

[5] Dadras SS (2011). Molecular diagnostics in melanoma: current status and perspectives.. Arch Pathol Lab Med.

[6] Ravo M, Mutarelli M, Ferraro L, Grober OM, Paris O (2008). Quantitative expression profiling of highly degraded RNA from formalin-fixed, paraffin-embedded breast tumor biopsies by oligonucleotide microarrays.. Lab Invest.

[7] Coudry RA, Meireles SI, Stoyanova R, Cooper HS, Carpino A (2007). Successful application of microarray technology to microdissected formalin-fixed, paraffin-embedded tissue.. J Mol Diagn.

[8] Cronin M, Pho M, Dutta D, Stephans JC, Shak S (2004). Measurement of gene expression in archival paraffin-embedded tissues: development and performance of a 92-gene reverse transcriptase-polymerase chain reaction assay.. Am J Pathol.

[9] Farragher SM, Tanney A, Kennedy RD, Paul Harkin D (2008). RNA expression analysis from formalin fixed paraffin embedded tissues.. Histochem Cell Biol.

[10] Linton KM, Hey Y, Saunders E, Jeziorska M, Denton J (2008). Acquisition of biologically relevant gene expression data by Affymetrix microarray analysis of archival formalin-fixed paraffin-embedded tumours.. Br J Cancer.

[11] Paik S, Shak S, Tang G, Kim C, Baker J (2004). A multigene assay to predict recurrence of tamoxifen-treated, node-negative breast cancer.. N Engl J Med.

[12] Penland SK, Keku TO, Torrice C, He X, Krishnamurthy J (2007). RNA expression analysis of formalin-fixed paraffin-embedded tumors.. Lab Invest.

[13] Devitt B, Liu W, Salemi R, Wolfe R, Kelly J (2011). Clinical outcome and pathological features associated with NRAS mutation in cutaneous melanoma.. Pigment Cell Melanoma Res.

[14] Long GV, Menzies AM, Nagrial AM, Haydu LE, Hamilton AL (2011). Prognostic and clinicopathologic associations of oncogenic BRAF in metastatic melanoma.. J Clin Oncol.

[15] Flaherty KT, McArthur G (2010). BRAF, a target in melanoma: implications for solid tumor drug development.. Cancer.

[16] Flaherty KT, Puzanov I, Kim KB, Ribas A, McArthur GA (2010). Inhibition of mutated, activated BRAF in metastatic melanoma.. N Engl J Med.

[17] Kim T, Kim J, Lee MG (2010). Inhibition of mutated BRAF in melanoma.. N Engl J Med.

[18] Poulikakos PI, Rosen N (2011). Mutant BRAF melanomas–dependence and resistance.. Cancer Cell.

[19] Solit DB, Rosen N (2011). Resistance to BRAF inhibition in melanomas.. N Engl J Med.

[20] Vitoux D, Mourah S, Kerob D, Verola O, Basset-Seguin N (2009). Highly sensitive multivariable assay detection of melanocytic differentiation antigens and angiogenesis biomarkers in sentinel lymph nodes with melanoma micrometastases.. Arch Dermatol.

[21] Mittempergher L, de Ronde JJ, Nieuwland M, Kerkhoven RM, Simon I (2011). Gene expression profiles from formalin fixed paraffin embedded breast cancer tissue are largely comparable to fresh frozen matched tissue.. PLoS One.

[22] Sanchez-Navarro I, Gamez-Pozo A, Gonzalez-Baron M, Pinto-Marin A, Hardisson D (2010). Comparison of gene expression profiling by reverse transcription quantitative PCR between fresh frozen and formalin-fixed, paraffin-embedded breast cancer tissues.. Biotechniques.

[23] Guedj M, Bourillon A, Combadieres C, Rodero M, Dieude P (2008). Variants of the MATP/SLC45A2 gene are protective for melanoma in the French population.. Hum Mutat.

[24] Chomczynski P, Sacchi N (1987). Single-step method of RNA isolation by acid guanidinium thiocyanate-phenol-chloroform extraction.. Anal Biochem.

