# Supplementary material for: A Reliable Method for the Selection of Exploitable Melanoma Archival Paraffin Embedded Tissues for Transcript Biomarker Profiling
Source: PLoS One. 2012 Jan 17;7(1):e29143. doi: 10.1371/journal.pone.0029143 (PMC3260139; doi:10.1371/journal.pone.0029143)
Supplement: Figure S1 — Classification approach: supplementary method defining the ‘Mean-Good-Expression-Profile’ (MGEP) as the mean of individual good samples expression profiles and those samples deviating significantly from the MGEP as bad sample profiles. Deviation was measured and tested with a classical chi-square test. (PDF) [file pone.0029143.s001.pdf]

Supp Methods 1: classification approach

Let n be the number of predictive reference genes.
Let μ_i and σ_i being respectively the mean and the standard deviation of the gene i assessed on the training set of good samples. It represents the 'Mean-Good-Expression-Profile' (MGEP) introduced in the main text.

For each new sample j to classify as 'good' or 'bad', let x_{ji} be its expression value for the gene i. We can then derive the following statistic measuring the deviation of the the sample j from the MGEP:

$$\mathcal{S}_j = \sum_1^n \left(\frac{x_{ji} - \mu_i}{\sigma_i} \right)^2 .$$

Under the null hypothesis of no difference (H_0), $\mathcal{S}_j$ follows a chi-square distribution with n degrees of freedom:

$$\mathcal{S}_j \underset{H0}{\sim} \chi^2(n).$$

It allows to compute a p-value and to define the following classification rule:
» if the p-value turns out non-significant, we conclude in no deviation from the MGEP, and classify the sample as 'good'.
» if the p-value turns out significant, we conclude in a substantial deviation from the MGEP, and classify the sample as 'bad'.

Further details on the implementation of this classification approach to our data are given in the main text.
